# Dual-Patch Technique with Ventricular Septal Defect Closure for Straddling Chordae

**DOI:** 10.1093/icvts/ivaf257

**Published:** 2025-10-24

**Authors:** Fumiya Yoneyama, Michiaki Imamura

**Affiliations:** Congenital Heart Surgery, Texas Children’s Hospital, Baylor College of Medicine, Houston, TX 77030, United States; Congenital Heart Surgery, Texas Children’s Hospital, Baylor College of Medicine, Houston, TX 77030, United States

**Keywords:** straddling valve, VSD closure, congenital heat surgery

## Abstract

Surgical repair of ventricular septal defects (VSDs) with straddling atrioventricular (AV) valve chordae is challenging due to the risk of disrupting valve integrity. We report the successful use of a dual-patch technique in a 5-month-old girl (6.1 kg) with Down syndrome, presenting with a large inlet VSD, secundum atrial septal defect (ASD), and straddling chordae involving both AV valves. Ventricular septal defects closure was performed via right atriotomy using 2 glutaraldehyde-treated autologous pericardial patches placed on the superior and inferior septal margins, encasing the chordae without division. Mitral and tricuspid valve clefts were repaired, and the ASD was closed primarily. Postoperative echocardiography showed no residual VSD and only mild AV valve regurgitation. This approach preserved valvular geometry and avoided conduction disturbance. The dual-patch technique offers a physiologic and conservative solution when conventional VSD repair is precluded by straddling chordae. It avoids chordal translocation or reimplantation, maintaining the native architecture and function of the AV valves.

## CASE REPORT

**Figure 1.**
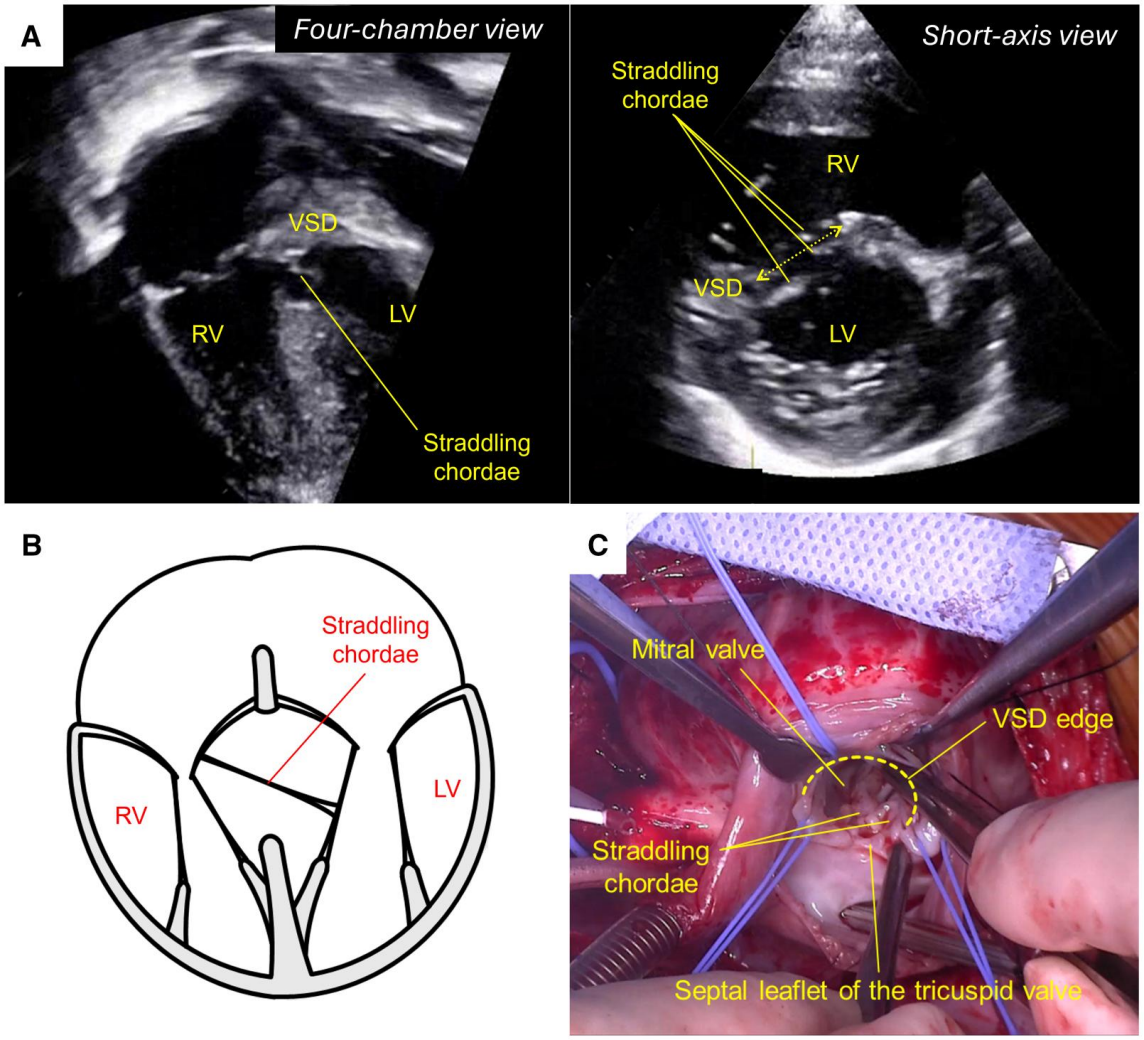
(A) Preoperative Echocardiography, (B) Straddling Chordae, and (C) Intraoperative Image. Abbreviations: LV, left ventricle; RV, right ventricle; VSD, ventricular septal defect

A 5-month-old girl (6.1 kg) with Down syndrome and a large ventricular septal defect (VSD) was referred for surgical repair. Preoperative echocardiography revealed a large inlet VSD, a small secundum atrial septal defect (ASD), clefts in both the mitral and tricuspid valves, and chordae crossing the VSD (**Figure 1A and C**).

Surgical repair was performed via median sternotomy under cardiopulmonary bypass (CPB) (**Video**). After systemic heparinization and initiation of CPB, the ductus arteriosus was ligated. Cardioplegic arrest was achieved, and the right atrium was opened. A large VSD extending from the inlet to the outlet septum was identified. Several chordae from the left ventricle inserting into both the tricuspid and mitral valves were observed (**Figure 1C**).

**Figure 2.**
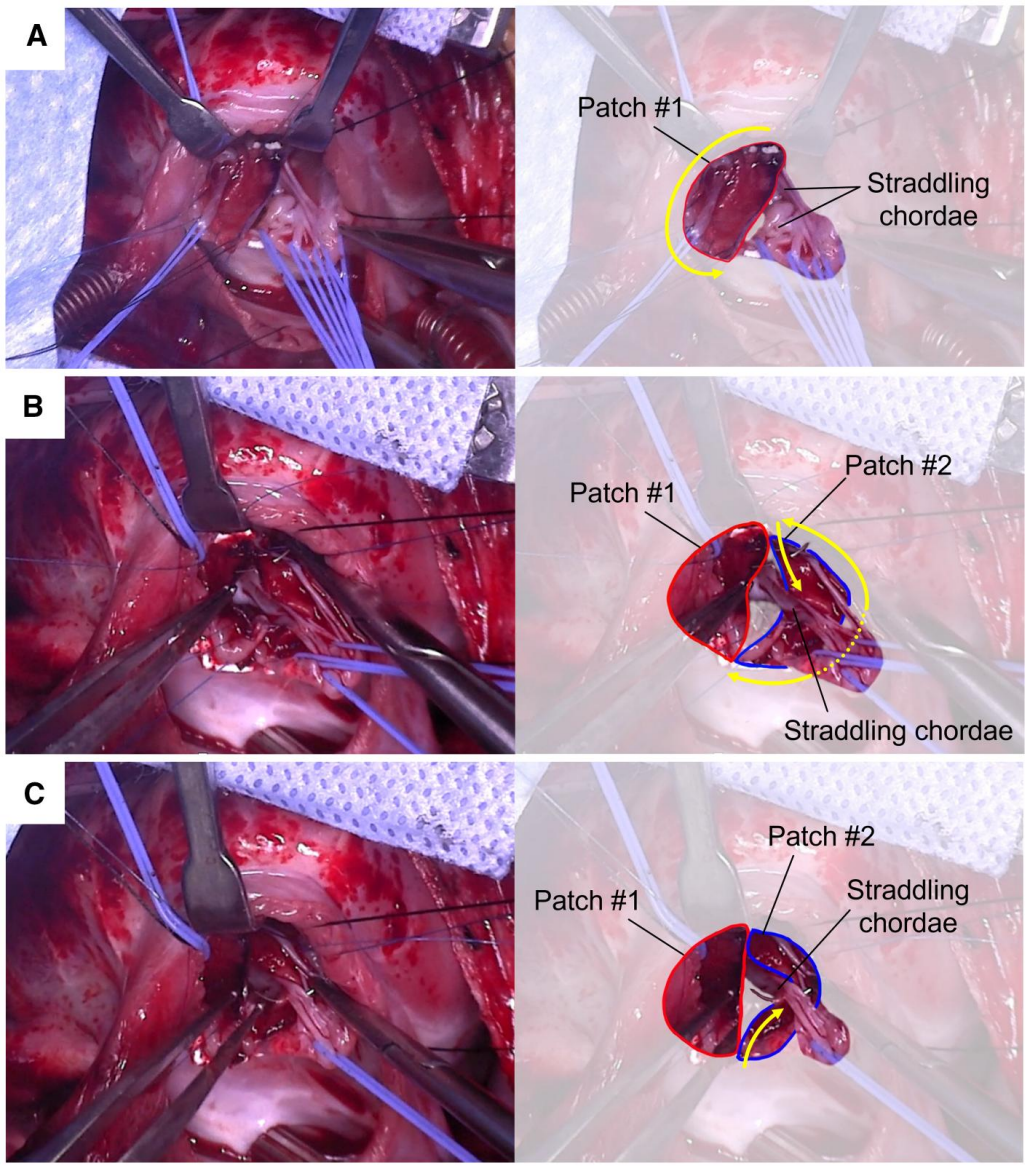
(A) The First Patch (Patch #1) was Placed on the Superior VSD Margin, Anchored to the Rim, Avoiding Interference with Straddling Chordae. (B) The Second Patch (Patch #2) was Applied Inferiorly to Encase the Straddling Chordae. The Anterior Gap between Patches Was Closed with Interrupted Sutures. (C) Final Approximation Closed the Posterior Gap, Ensuring the Chordae Remained Protected Without Distortion or Tension. Abbreviation: VSD, ventricular septal defect

To preserve these chordae, a dual-patch technique was employed. Two glutaraldehyde-treated autologous pericardial patches were used to encase the chordae during VSD closure. The first patch was applied to the superior VSD margin (**Figure 2A**), followed by the second patch on the inferior side, carefully avoiding the inferior rim to protect the conduction system (**Figure 2B**). Both patches were secured with pledgeted 6–0 Prolene interrupted and running sutures. After confirming proper chordal positioning, the two patches were approximated and joined with interrupted 6–0 mattress sutures (**Figure 2C**).

Valvuloplasty was performed for both AV valves due to clefts. The mitral cleft edges appeared dysplastic; partial cleft closure was performed at the base using 6–0 Prolene mattress sutures. The tricuspid valve, which showed regurgitation through a cleft-like defect on the septal leaflet, was repaired with multiple interrupted 6–0 Prolene sutures. The enlarged ASD was closed with 5–0 Prolene sutures.

After de-airing, the aortic cross-clamp was removed. The heart resumed sinus rhythm. The postoperative course was uneventful, and echocardiography on postoperative day 3 showed good function, no significant residual VSD, mild tricuspid valve regurgitation, and trivial mitral valve regurgitation.

## DISCUSSION

Surgical repair of straddling valves associated with VSDs remains technically demanding. These rare anomalies are characterized by chordae tendineae or papillary muscles that cross the interventricular septum and insert into both ventricles. Straddling is typically classified into three types (I–III) based on anatomical configuration and the extent of ventricular involvement.^1^

Conventional surgical strategies include chordal detachment, translocation, and realignment.^2^^,^^3^^,^^4^ However, these approaches require manipulation of the subvalvular apparatus and may alter native valvular geometry. In particular, when straddling chordae share a papillary muscle with the opposing AV valve, as in our case, dividing or repositioning these structures may compromise the functional integrity of both valves.

To address this challenge, we employed a dual-patch technique that preserves the straddling chordae in situ. By encasing the chordae between 2 patches, this method maintains continuity of the leaflet—chordae—papillary muscle complex. It also avoids direct incision or reimplantation of chordal structures, which is especially advantageous in cases involving multiple straddling chordae, where individualized translocation may be technically demanding.

Tailored patch placement also enables precise VSD closure while minimizing the risk of conduction system injury. In hearts with straddling AV valves, the conduction tissue may be variably displaced due to septal malalignment or abnormalities near the crux of the heart.^5^ Surgical manipulation in these areas—particularly involving chordal reattachment or translocation—can increase the risk of conduction disturbances. In contrast, the dual-patch technique enables closure without disturbing these vulnerable regions. In our case, this method resulted in successful VSD closure without conduction disturbance and preserved valve competence. Long-term follow-up remains essential to monitor AV valve function, given the underlying straddling and complex anatomy. The dual-patch technique thus offers a physiologic and structurally conservative alternative in selected patients with straddling AV valve anatomy.

## Data Availability

All data underlying this article are included within the manuscript.
